# MiR-152 Regulates Apoptosis and Triglyceride Production in MECs *via* Targeting *ACAA2* and *HSD17B12* Genes

**DOI:** 10.1038/s41598-017-18804-x

**Published:** 2018-01-11

**Authors:** Yuwei Yang, Xibi Fang, Runjun Yang, Haibin Yu, Ping Jiang, Boxing Sun, Zhihui Zhao

**Affiliations:** 1 Agricultural College, Guangdong Ocean University, Zhanjiang, 524088 China; 2 College of Animal Science, Jilin University, Xi An Road 5333, Changchun, Jilin 130062 P.R. China

## Abstract

Mammary epithelial cells (MECs) affect milk production capacity during lactation and are critical for the maintenance of tissue homeostasis. Our previous studies have revealed that the expression of miR-152 was increased significantly in MECs of cows with high milk production. In the present study, bioinformatics analysis identified *ACAA2* and *HSD17B12* as the potential targets of miR-152, which were further validated by dual-luciferase repoter assay. In addition, the expressions of miR-152 was shown to be negatively correlated with levels of mRNA and protein of *ACAA2*, *HSD17B12* genes by qPCR and western bot analysis. Furthermore, transfection with miR-152 significantly up-regulated triglyceride production, promoted proliferation and inhibited apoptosis in MECs. Furthermore, overexpression of *ACAA2* and *HSD17B12* could inhibit triglyceride production, cells proliferation and induce apoptosis; but *sh234-ACAA2-181/sh234-HSD17B12-474* could reverse the trend. These findings suggested that miR-152 could significantly influence triglyceride production and suppress apoptosis, possibly via the expression of target genes *ACAA2* and *HSD17B12*.

## Introduction

Breast fatty gland tissues not only help to support the basic mammary structure, but also serve as the communication bridge between mammary epithelia and their local and systemic environment throughout the development of breast^1^, which are mainly consisted of a large number of mammary epithelial cell clusters. Cow has four mammary glands in each breast which are responsible for the synthesis and secretion of milk proteins^2^. More importantly, the growth and development of mammary epithelial cells affect milk production capacity during lactation and new mammary epithelial cells formation is critical for maintenance of tissue homeostasis^3^.

MicroRNA (miRNA) as an mRNA suppresser regulates protein quantity and controls a variety of cellular and physiological processes by post-transcriptional processing, including cell proliferation, differentiation, apoptosis and tumor metastasis^4–9^. Recent studies have shown that miRNAs are involved in fat formation and lipolysis^10–14^. Certain miRNAs have been reported to either promote or inhibit triglyceride production and to suppressapoptosis of MECs at growing stages. For instance, miR-224 overexpression could decrease triglyceride synthesis and promote apoptosis^12^. Bta-miR-29b promotes triglyceride production and suppress apoptosis^11^. In addition, miR-33a, miR-21, miR-23a, miR-877 have also been reported to affect triglyceride production and apoptosis of MECs^10^. Nonetheless, the effects of miR-152 expression on MECs have not been well studied.

The mature sequence of miR-152, a member of the miR-148/152 family (miR-148a, miR-148b, and miR-152), is relatively conservative^15^. Previous studies on miR-152 are mostly focused on tumor. It has been reported that miR-152 might be involved in the carcinogenesis of ovarian cancer through deregulation of cell proliferation and might be a novel biomarker for early detection or therapeutic purpose^16^. MiR-152 affects cell cycle progression in non-small cell lung cancer and liver cancer by targeting WNT-1 protein^17^. There are also evidences suggest that miR-152 is associated with epigenetics. MiR-152 and miR-185 could co- target DNMT1 (DNA Methyltransferase 1) in ovarian cancer cells^18,19^. In recent years, increased studies indicate that miR-152 is involved in lipid metabolism, for example, miR-152 regulates DNMT1, which in turn influences lactation-related genes in dairy cow mammary epithelial cells^20^. However, miR-152 has not been comprehensively investigated in MECs, especially the involvement of miR-152 in the mammary gland at the molecular level.

MiRNA could control gene expression either via target mRNAs degradation or translation inhibition by binding to the 3′-untranslated regions (*3-UTR*)^19,21,22^. Bioinformatics analysis suggest that *ACAA2* (acetyl-CoA acyltransferase 2) and *HSD17B12* (hydroxysteroid 17-beta dehydrogenase 12) are both potential target genes of miR-152.

Acetyl-CoA acyltransferase 2 (*ACAA2*) gene encodes an enzyme of the thiolase family, which is involved in mitochondrial fatty acid elongation and degradation by catalyzing the last step of the respective β-oxidation pathway^23^. Meanwhile, BNIP3, a unique pro-apoptotic protein, belonging to the BH3-only subset of the Bcl-2 family, was found to have a linkage with *ACAA2*. Cell apoptosis from overexpression of BNIP3 or hypoxia treatment could be abolished by *ACAA2* expression^24^. The Hydroxysteroid (17b) dehydrogenase type 12 (*HSD17B12*) gene belongs to the hydroxysteroid (17) dehydrogenase superfamily. *HSD17B12* encodes a multifunctional enzyme, which is involved in the prolongation of very long chain fatty acid (VLCFA), especially in the conversion of palmitic to archadonic (AA) acid and in the synthesis of arachidonic acid (AA)^25–27^.

The present study aims to identify the role of miR-152 in mammary epithelial cells. Our results suggested that miR-152 could regulate fatty acid metabolism by directly targeting *ACAA2*, *HSD17B12*, which in turn inhibited cellular apoptosis, promoted cell proliferation and enhanced triglyceride production. Further study of miR-152 in lipid metabolism and apoptosis in MECs might establish this microRNA as a novel biomarker for marker assisted selection of Holstein dairy cows, especially in the selection of butterfat rate.

## Results

### *ACAA2* and *HSD17B12* were the direct target genes of miR-152 which were verified in MECs

**Figure 1:**
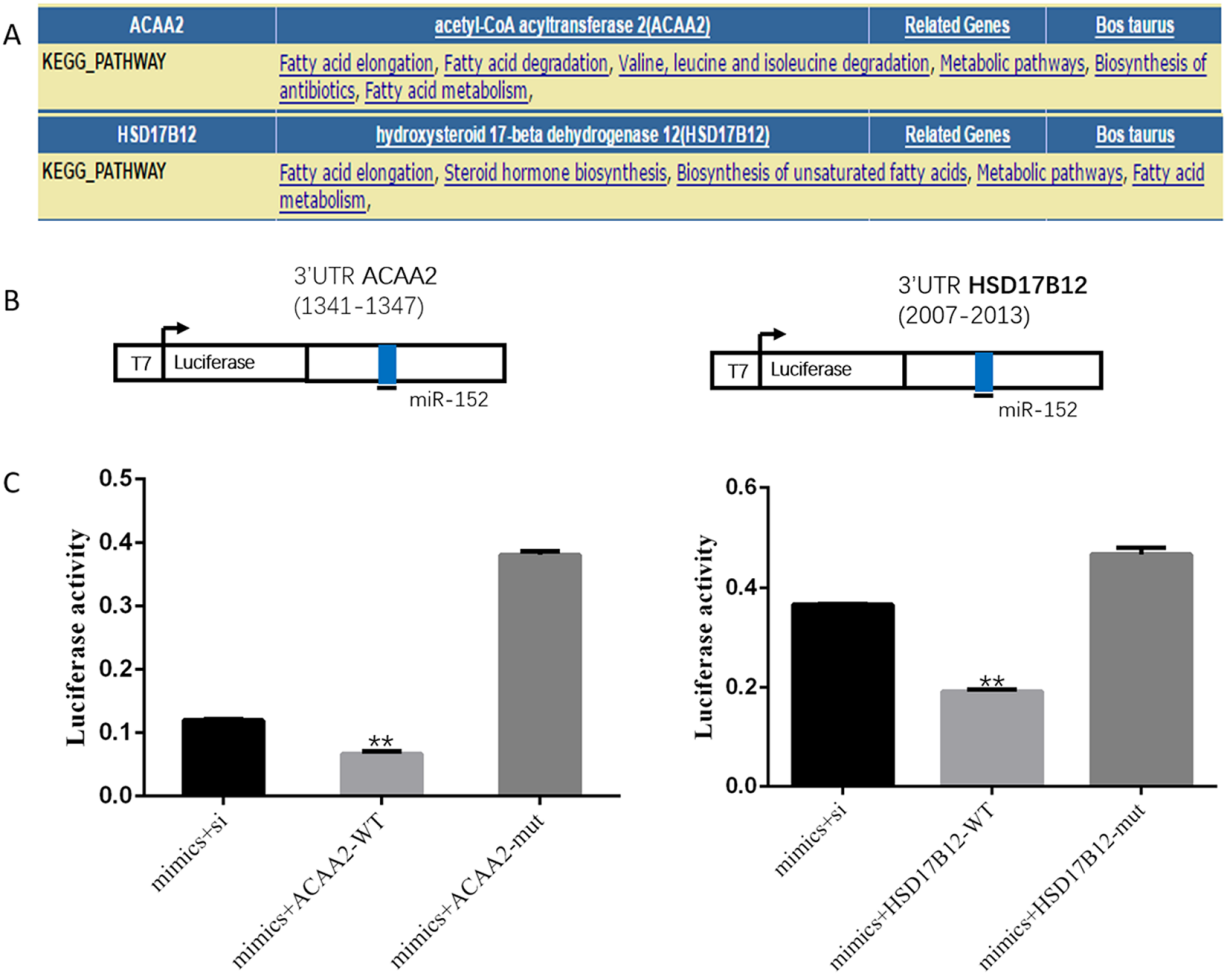
Target sites of miR-152 and luciferase assay. (**A**) The pathways of *ACAA2* and *HSD17B12* were analyzed using the DAVID system. (**B**) Binding sites of miR-152 on the 3′UTR of *ACAA2* and *HSD17B12*. (**C**) Luciferase activities were detected in MECs co-transfected with miR-152 and *pmiR-RB-REPORT-ACAA2-mut/WT/NC* vector or *pmiR-RB-REPORT- HSD17B12-mut/WT/NC* vector. (**p < 0.01).

Many target genes of miR-152 were predicted by high-throughput sequencing. Among all the potential genes, *ACAA2* and *HSD17B12* were selected for further validation due to their potential roles in fatty acid and triglyceride formation using DAVID system (Fig. 1A). Both *ACAA2* and *HSD17B12* genes had one miR-152 binding sites in 3′UTR region. (Fig. 1B). Co-transfection results showed that luciferase activities of MECs transfected with *ACAA2-WT* or *HSD17B12-WT* vector significantly decreased compared with MECs transfected with *ACAA2-si/mut* vector and *HSD17B12/mut* vector (Fig. 1C). These results further confirmed that *ACAA2* and *HSD17B12* were target genes of miR-152.

### *ACAA2 and HSD17B12* were down-regulated by miR-152

**Figure 2:**
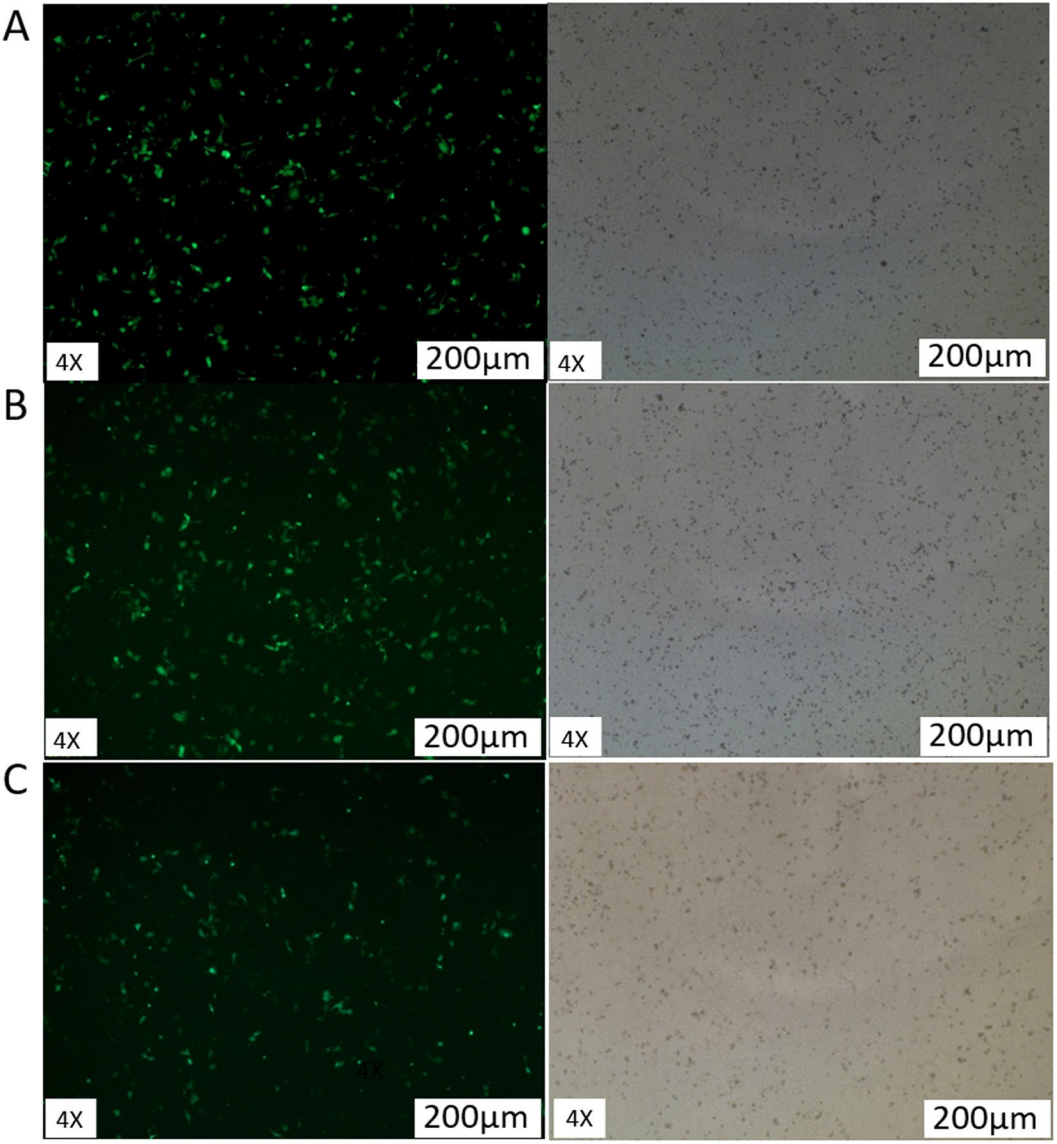
MEC transfection efficiency. Green fluorescence could be observed under a fluorescence microscope 24 h after the transfection. The expression rate of green fluorescence in mammary epithelial cells of dairy cow cells was 60%. (**A**) Cells transfected with *miR-152 mimics*; (**B**) Cells transfected with *miR-152 inhibitor*; (**C**) Cells transfected with *miR-shNC*.

**Figure 3:**
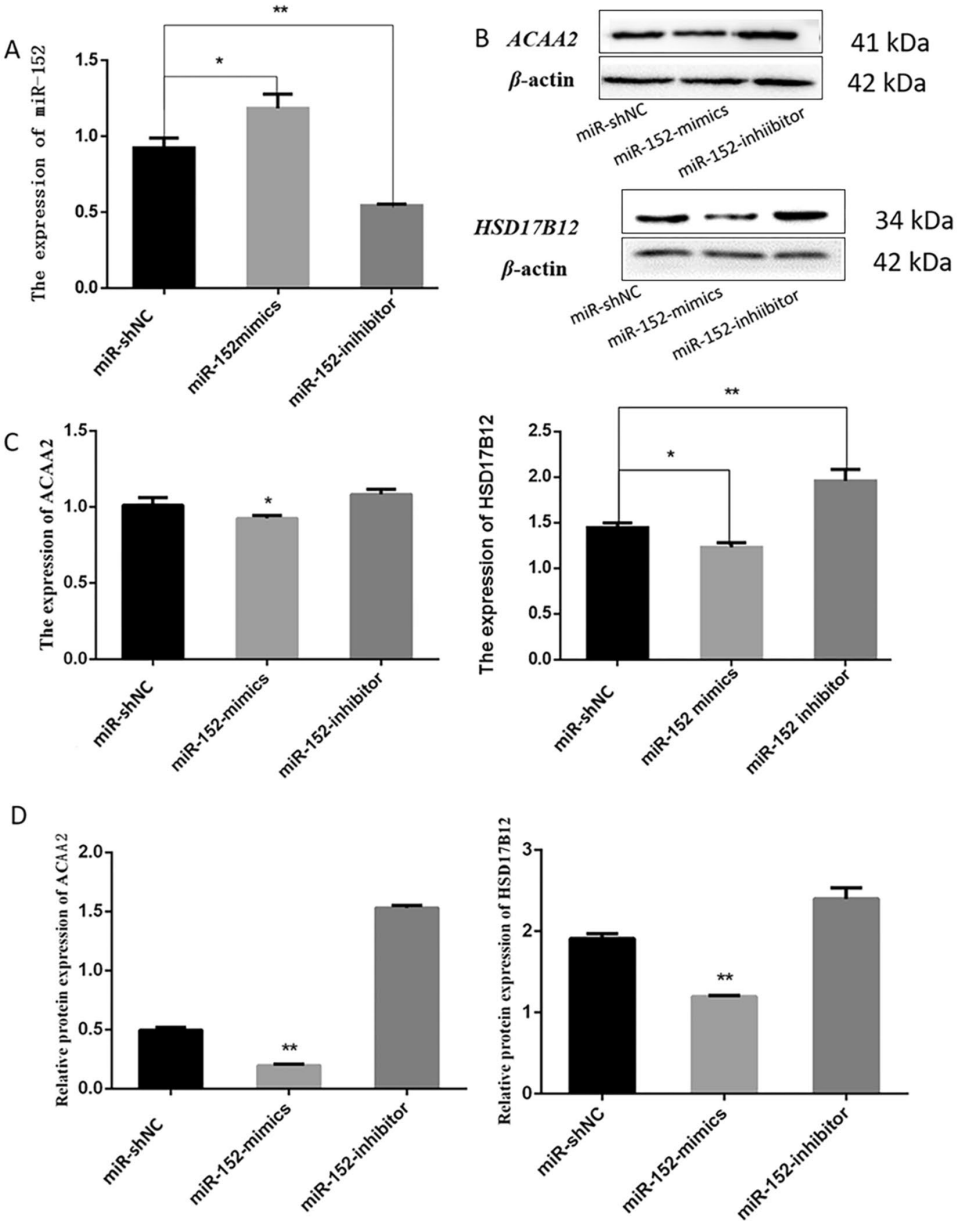
The expressions of miR-152b and target genes. (**A**) qPCR analysis of the expressions of miR-152 in MECs transfected with *miR-152 mimics*, *miR-152 inhibitor* and *miR-shNC*. (**B**) Western blot analysis of the protein levels of *ACAA2* and *HSD17B12* genes in MECs transfected with *miR-152 mimics*, *miR-152 inhibitor* and *miR-shNC*. (**C**) qPCR analysis for the mRNA levels of *ACAA2* and *HSD17B12* genes in MECs transfected with *miR-152 mimics*, *miR-152 inhibitor* and *miR-shNC*. (**D**) Relative levels of *ACAA2* and *HSD17B12* protein in MECs transfected with *miR-152 mimics*, *miR-152 inhibitor* and *miR-shNC*. (**p < 0.01, *p < 0.05).

To test whether miR-152 down-regulates the expression of *ACAA2* and *HSD17B12*, *miR-152 mimics* and *miR-152 inhibitor* were transfected into MECs and the expressions of GFP protein can be observed in cells transfected with *miR-152 mimics*, *miR-152 inhibitor* and *miRNA-ShNC* (Fig. 2), suggesting that the transfection was successful. The expressions of *ACAA2* and HSD*17B12* at mRNA and protein level were examined (Fig. 3B,C and D) (Fig. S1.1). The results showed that the expression levels of miR-152 were remarkably increased in MECs transfected with miR-152 *mimics* as compared with that transfected with miR-152 inhibitor and *miRNA-shNC* (Fig. 3A). Moreover, transfection of *miR-152 mimics* significantly decreased the expressions of *ACAA2* and *HSD17B12* at both mRNA and protein levels, indicating that miR-152 down-regulated the expression of both *ACAA2* and *HSD17B12* genes.

### MiR-152 promoted cell proliferation and inhibited apoptosis

**Figure 4:**
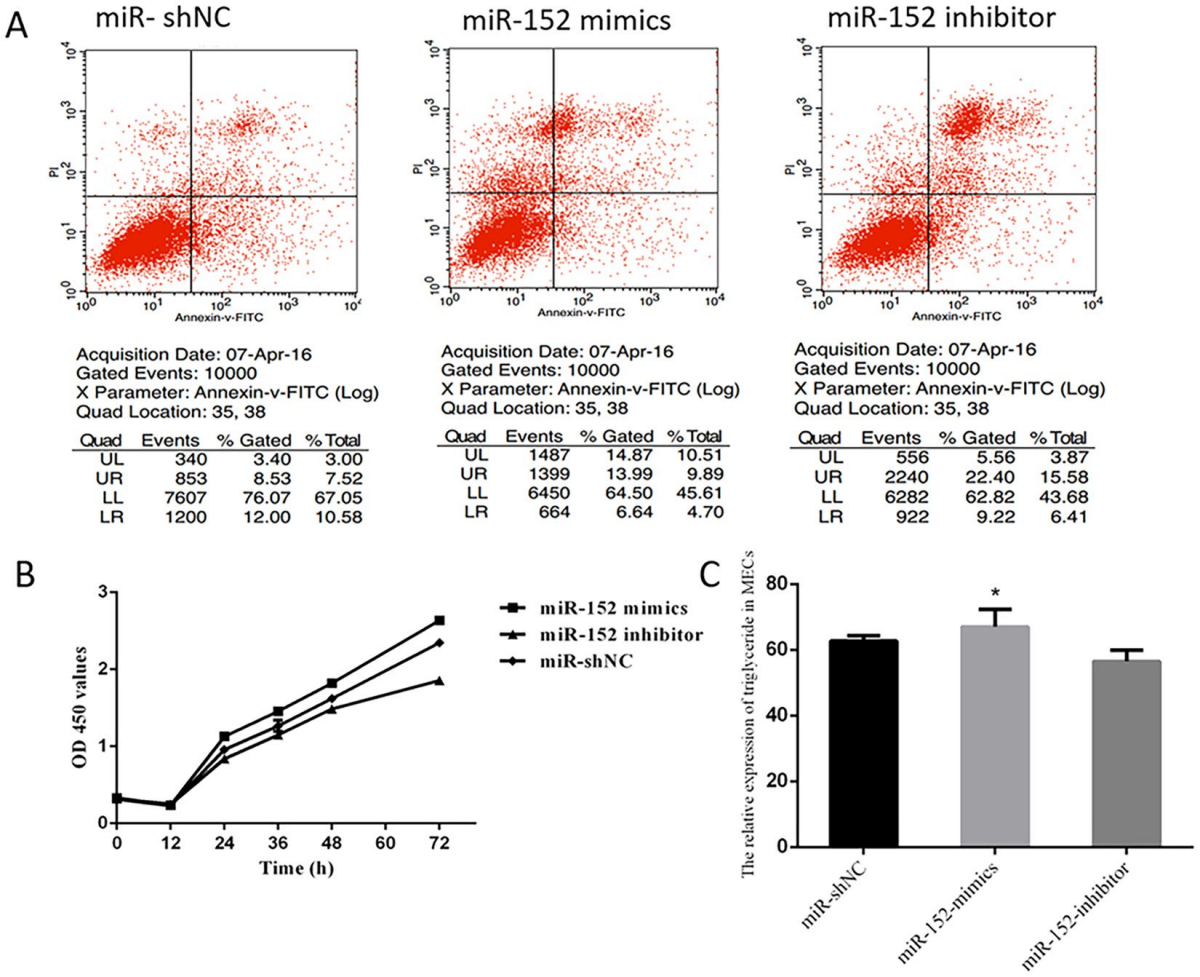
miR-152 effect on cell apoptosis, cell proliferation and triglycerides production in MECs. (**A**) Apoptosis ratio of MECs transfected with *miR-152 mimics*, *miR-152 inhibitor* and *miR-shNC*. (**B**) MECs proliferation determined by the MTT assay in MECs transfected with *miR-152 mimics, miR-152 inhibitor* and *miR-shNC*. (**C**) Triglycerides level in MECs transfected with *miR-152 mimics*, *miR-152 inhibitor* and *miR-shNC*. (*p < 0.05).

The apoptosis rate of MECs transfected with the *miR-152 mimics*, *miR-152 inhibitor* and *miRNA-shNC* were examined using flow cytometry. The results showed that the apoptosis rates were 14.59%, 21.99% and 18.1% in MECs transfected with the *mimics*, *inhibitor* and *shNC* of miR-152, respectively (Fig. 4A), suggesting that overexpression of miR-152 inhibited the apoptosis of MECs.

The cellular proliferation rate was determined by an MTT assay after the cells were transfected with *miR-152 mimics*, *miR-152 inhibitor*, *miRNA-ShNC* and cultured for 0, 12, 24, 36, 48 or 72 h. As shown in Fig. 4A, *miR-152 mimics* induced a significant decrease on the proliferation rate of MECs (p < 0.01) (Fig. 4B).

### Regulation of triglyceride production by miR-152

Triglyceride production was upregulated in MECs transfected with *miR-152 mimics*, and no significant differences were observed in MECs transfected with *inhibitor*, *shNC* and *blank*, respectively (Fig. 4C) (p < 0.05).

### *ACAA2* and *HSD17B12* effect on cell apoptosis, proliferation and triglyceride production

*PBI-CMV3-ACAA2/HSD17B12* and *sh234-ACAA2-181/sh234-HSD17B12-474* plasmids were respectively transfected into cells using lipofectamine TM 2000. The apoptosis rates were detected as follows: *PBI-CMV3-ACAA2* (7.49%), *PBI-CMV3* (4.89%), *sh234-ACAA2-181* (7.08%), *sh234* (6.62%), *PBI-CMV3-HSD17B12* (8.9%), and *sh234-HSD17B12-474* (2.58%) respectively. The results showed that overexpression of *ACAA2* and *HSD17B12* could induce cells apoptosis; but *sh234-ACAA2-181/sh234-HSD17B12-474* could reverse the effect.

After MTT detection, cells proliferation ability was better in group of *sh234-ACAA2-181/sh234-HSD17B12-474* transfection than cells transfected *PBI-CMV3-ACAA2/HSD17B12* plasmids.

**Figure 5:**
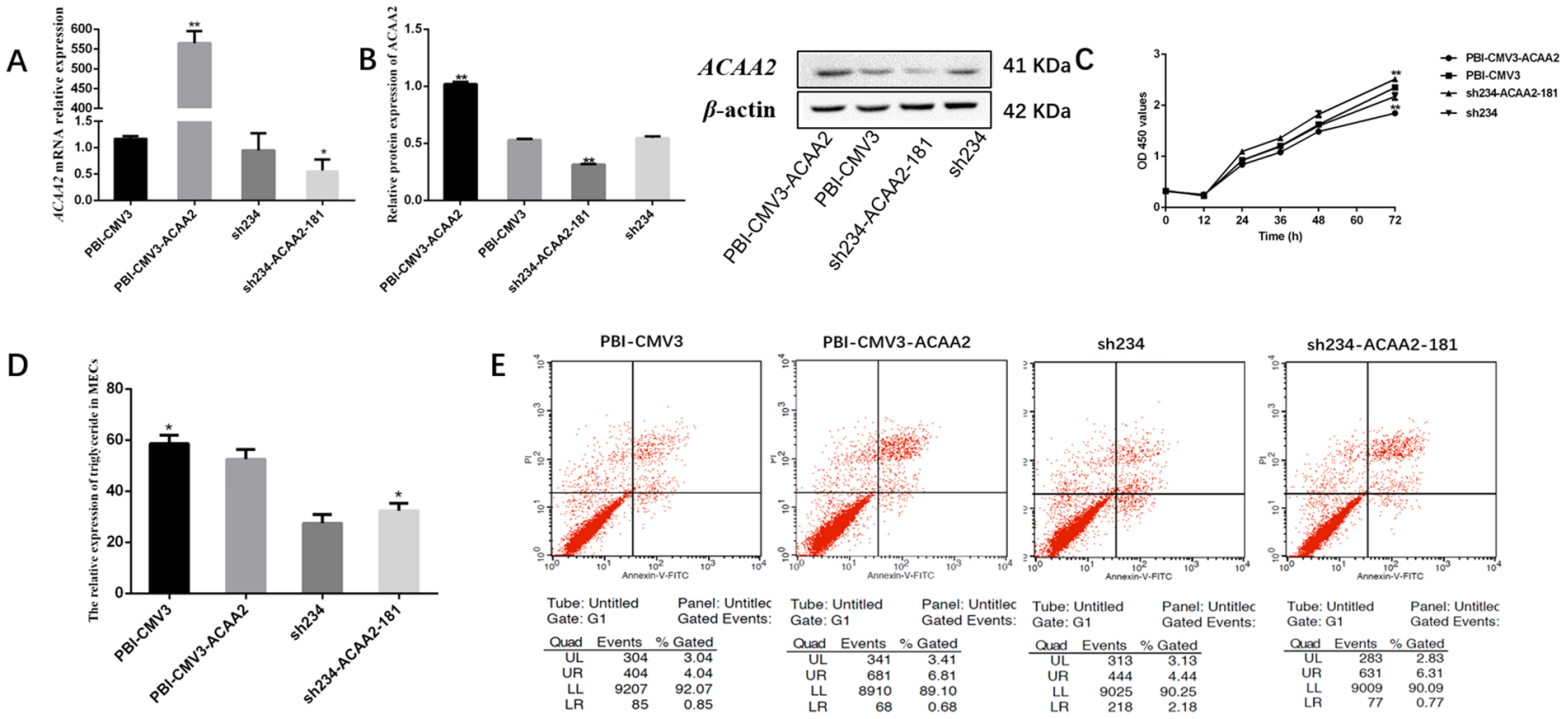
*ACAA2* gene effect on cell apoptosis, proliferation and triglyceride production. (**A**) *PBI-CMV3-ACAA2* and *sh234-ACAA2-181* plasmids were respectively transfected into cells. Then qPCR analysis of the expressions of *ACAA2*. (**B**) Proteins of ACAA2 in cells after transfection. (**C**) MECs proliferation were analyzed after *PBI-CMV3-ACAA2* and *sh234-ACAA2-181* plasmids treatment. (**D**) triglyceride production in MECs. (**E**) Apoptosis rate was detected in MECs transfected with *PBI-CMV3-ACAA2* and *sh234-ACAA2-181*.

**Figure 6:**
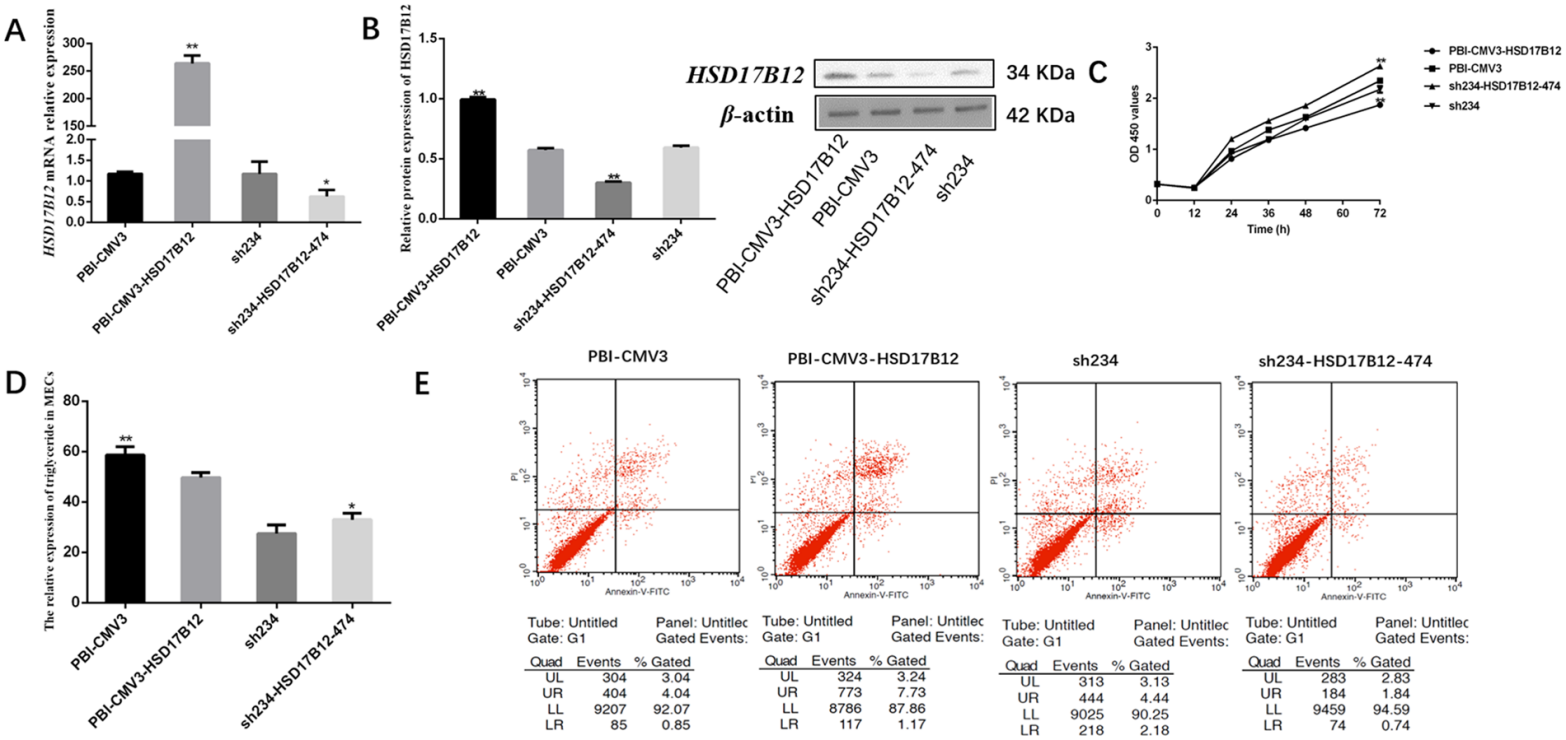
*HSD17B12* gene effect on cell apoptosis, proliferation and triglyceride production. (**A**) *HSD17B12* mRNA detected in MECs that transfected *PBI-CMV3-HSD17B12* and *sh234-HSD17B12-474* plasmids. (**B**) Proteins of *HSD17B12* were analyzed by western blot in MECs. (**C**) MTT was used to assay the effect of *HSD17B12* on MECs proliferation ability. (**D**) Influence of *HSD17B12* on triglyceride was assessed. (**E**) Apoptosis rate were analyzed after plasmids transfection.

In addition, overexpression of *ACAA2* and *HSD17B12* could inhibit triglyceride production. But *sh234-ACAA2-181/sh234-HSD17B12-474* could improve the expression (Figs 5 and 6).

## Discussion

Triglyceride, the main form of energy storage in the body, is stored in adipose tissue which is involved in a range of processes such as energy balance and fatty acids metabolism. Triglycerides are ester molecules derived from glycerol and three fatty acids^28–31^. Apoptosis is a process of programmed cell death that occurs in multicellular organisms^32,33^, which involve coordinated regulation of a broad range of genes.

Here we identified the regulatory roles of miR-152 on *ACAA2* and *HSD17B12* in triglyceride production and apoptosis of MECs. Our results suggested that miR-152 represses the expression of *ACAA2* and *HSD17B12* through a direct interaction with the 3′*UTR* regions of the *ACAA2* and *HSD17B12*. When RNA is bound by miRNAs, miRNA-induced silencing complex is thought to inhibit protein production either through blocking translation or by reducing messenger RNA stability^34^. Our results also indicated that miR-152 could suppress the expression of *ACAA2* and *HSD17B12* by reducing the stability of mRNA.

Furthermore, in our study, overexpression of *ACAA2* and *HSD17B12* could inhibit triglyceride production, cells proliferation and induce apoptosis; but *sh234-ACAA2-181/sh234-HSD17B12-474* could reverse the trend. Gene pathway analysis suggests that upregulation of *ACAA2* and *HSD17B12* could induce fatty acid elongation. In mammals, *HSD17B12* is involved in lipid metabolism^35^, such as in the synthesis of arachidonic acid. *HSD17B12* is also essential for normal neuronal development during embryogenesis^36^. *ACAA2* encodes an enzyme of the thiolase family that is involved in fatty acid elongation and degradation by catalyzing the last step of the respective β-oxidation pathway. The increased energy needs for gluconeogenesis and triglyceride synthesis during lactation are met primarily by increased fatty acid oxidation. Therefore, ACAA2 enzyme plays an important role in the supply of energy and carbon substrates for lactation and may thus affect milk production traits^23^. In addition, the viability and apoptosis of MECs is also a key factor affecting lipid metabolism. Several recent studies show that tumor cell growth was inhibited and apoptosis was increased upon silencing of *HSD17B12* ^37,38^. ACAA2 has also been shown to be a functional BNIP3 binding partner, which provides a possible link between fatty acid metabolism and cell apoptosis. *ACAA2* counteracts the apoptosis induced by BNIP3. BNIP3 is a unique pro-apoptotic protein which belongs to the BH3-only subset of the Bcl-2 family and localizes on mitochondrial membrane in HepG2 cells^24^.

It has been reported that simultaneous inhibition of miR-148a and miR-152 could significantly protect MCF-7 cells from 4-OHT induced cell viability reduction and inhibit cell apoptosis^39^. MiR-152-3p might also act as a tumor suppressor in human breast cancer cells via negatively regulating *PIK3CA* expression to inhibit the activation of *AKT* and *RPS6*, leading to the suppression of HCC1806 cells proliferation^40^. MiR-152 inhibits tumor cell growth by directly targeting *RTKN* in hepatocellular carcinoma^41^. In addition, miR-152 could enhance the viability and multiplication capacity of DCMECs^20^. Similarly, our results showed that miR-152 could inhibit apoptosis and promote cell proliferation in MECs. In summary, *ACAA2* and *HSD17B12*, two important genes involved in lipid metabolism, were targeted and regulated by miR-152. In addition, miR-152 could influence mammary epithelial cells apoptosis and triglyceride formation. Therefore, miR-152 could be considered as an important indicator for evaluation of cow’s milk fat quality and marker assisted cattle breeding.

## Materials and Methods

The experimental design and procedures were performed in accordance with the approved Guidelines for Animal Experiments of Jilin University, China and were approved by the Animal Care and Use Committee of Jilin University, China (Approval ID: SYXK (Ji) 2008-0010/0011).

### Experimental Reagents

The primers were synthesized by Shanghai Sangon Biotech Company in China. The RNA Extraction Kit, cDNA reverse transcription Kit and the SYBR Green were from Takara (Takara Biological Company, Japan). *ACAA2* antibody and *HSD17B12* antibody were from abcam (abacm Reagent Company, USA). The Dual luciferase assay kit was from Promega (Promega Company, USA).

### Target Prediction and KEGG Orthology Analysis

Based on the sequences of the miRNAs, target genes were predicted by deep sequencing and screening. The KEGG of all small RNA target genes was analyzed using DAVID system (https://david.ncifcrf.gov/home.jsp). The predicted target genes were subsequently submitted to KOBAS for KEGG Orthology analysis (http://kobas.cbi.pku.edu.cn/home.do) using KEGG database.

### Plasmid Construction

Vectors of *miR-152 mimics*, *miR-152 inhibitor* and *miR-shNC* were purchased from GenePharma Company in China. The sequences of *ACAA2* and *HSD17B12* were amplified with PCR instrument with *Not* I and *Xho* I restriction sites engineered at primer ends. Amplified DNA fragments were cloned into *pmiR-RB-REPORT* vector to construct recombinant vectors *pmiR-RB-REPORT- ACAA2-mut/WT/si and pmiR-RB-REPORT- HSD17B12-mut/WT/si*. To investigate the association between target genes with apoptosis, cell proliferation and triglyceride production, *PBI-CMV3-ACAA2/HSD17B12* and *sh234-ACAA2-181/sh234-HSD17B12-474* plasmids were respectively constructed, and then transfected into cells using lipofectamine TM 2000 according to the manufacturer’s instructions.

### Cells culture and Transfection

6 heads of healthy cows were selected, then mammary epithelial cells was isolated from dairy cows with high-fat production. Mammary epithelial cells was isolated and cultured from the Laboratory of animal genetics in Jilin University. Progesterone was added into the basal medium. After determination of cell viability and concentration, cells were seeded in six-well culture plates (Corning Inc., Corning, NY) the day before transfection at a density of approximately 1 × 10^6^ per well with DMEM/F12 (GIBCO, Grand Island, NY, USA) containing 10% fetal bovine serum (FBS; PAA, Pasching, Austria) and incubated at 37 °C in a 5% CO_2_ incubator with humidified atmosphere.

For luciferase activity detection, 150 µL Opti-Minimal Essential Medium (MEM) serum-free medium (GIBCO, Grand Island, NY, USA) was mixed with 5 µL lipofectamine TM 2000 (Invitrogen, USA) and 1.25 µL 20 µmol of the miR-152 mimics and 500 ng *pmiR-RB-REPORT* vectors. To validate the miR-152 effects on the target genes and triglyceride production, 150 µL Opti-MEM serum-free medium was mixed with 5 µL of lipofectamine TM 2000 and 1.25 µL 20 µmol of the *miR-152 mimics*, *inhibitor* and *miR-shNC*. To investigate the association between target genes with apoptosis and triglyceride production, *PBI-CMV3-ACAA2/HSD17B12* and *sh234-ACAA2-181/sh234-HSD17B12-474* plasmids were respectively transfected into cells using lipofectamine TM 2000 according to the manufacturer’s instructions.

### Luciferase Reporter Assay

MECs were maintained in DMEM/F12 (GIBCO) supplemented with 10% fetal bovine serum (FBS, GIBCO). The cells were transiently co-transfected with 0.5 μg of reporter plasmids (*pmiR-RB-REPORT-ACAA2-mut/WT/si or pmiR-RB-REPORT-HSD17B12-mut/WT/si*) and *miR-152 mimics*. The activity of luciferase was detected by using the SpectraMax M5 Microplate Reader.

### Real-time PCR Analysis

qRT-PCR was utilized to measure the expression levels of miRNAs and mRNAs. The reverse transcription primers and fluorescence labeled primers for quantitative analysis of miR-152 and target genes were designed using Primer 6.0 as shown in Table 1. MECs transfected with *miR-152 mimics*, *miR-152 inhibitor* and *miRNA-ShNC* were harvested at 48 h post-transfection and total RNA from cultured cells was extracted by using TRIzol reagent (Ambion, Austin, TX, USA) according to the manufacturer’s instructions. The cDNA was synthesized with the Reverse Transcription Kit. The expression levels of miRNA and mRNA were assessed with qRT-PCR using SYBR Green I (Takara, Dalian, China) on an Eppendorf AG-5341 instrument. Three biological repeats and three technical replicates were measured. The expression levels of miRNA and mRNA were defined by the following formula:

$${\rm{\Delta }}{\rm{\Delta }}\mathrm{Ct}=\{\mathrm{Ct}(\mathrm{positive})-\mathrm{Ct}(\mathrm{reference})\}-\{\mathrm{Ct}(\mathrm{control})-\mathrm{Ct}(\mathrm{reference})\}.$$

Here, 2−ΔΔCt refers to the relative expression ratio and relative expression levels were calculated using the 2 −ΔΔCt method. The expression levels of U6 small nuclear RNA was used as housekeeping gene of miR-152, and GAPDH was used as reference genes of target genes individually.

**Table 1:** Primer sequences of real-time PCR.

Symbol	Primer	Primer Sequence (5′-3′)	Amplicon size
Bta-miR-152	RT-Primer	GTCGTATCCAGTGCAGGGTCCGAGGTATTCGCACTGGATACGACCCCAAGTT	22 bp
	F-Primer	TGCGGTCAGTGCATGACAGAA	
	R-Primer	CAGTGCAGGGTCCGAGGT	
U6	RT-Primer	CGCTTCACGA ATTTGCGTGTCAT	20 bp
	F-Primer	GCTTCGGCAGCACATATACTAAAAT	
	R-Primer	CGCTTCACGA ATTTGCGTGTCAT	
*ACAA2*	F-Primer	TAATGAGATGGCACCAGTTGAGG	200 bp
	R-Primer	AGCACCATCGGAAACCCC	
*HSD17B12*	F-Primer	CCTACCTGGCACTGCGTATCTCA	183 bp
	R-Primer	CATTCCACGCTCTGCTAACTTTTCT	
*GAPDH*	F-Primer	ATTCTGGCAAGTGGACATCG	431 bp
	R -Primer	ACATACTCAGCACCAGCATCAC	

### Western Blot Analysis

Cultured cells were lysed in RIPA buffer (Boster, Wuhan, China) with 1% Phenylmethanesulfonyl fluoride (Beyotime, China) following the manufacturer’s instruction. Protein was loaded and seperated by SDS-PAGE gel and transferred onto PVDF membrane (Bio-Red Laboratories Inc, USA). Then, the polyvinylidene difluoride (PVDF) membrane were incubated with *anti-ACAA2* (1:1000, Abcam, Cambridge, MA, USA), *anti-HSD17B12* (1:1000, Abcam) or *anti-actin* (1:1000, Abcam) at 4 °C overnight. After washing 3 times by Tris Buffered Saline with Tween 20 (TBST), the PVDF membrane was incubated with goat anti-rabbit IgG (1:3000, Abcam) for 1 h at room temperature. Finally, protein bandings were obtained by enhanced chemiluminescence (ECL) Western Blotting Substrate (Invitrogen USA). And the signal intensities were captured by a Tanon 5200 chemiluminescence/fluorescence image analysis system. *β-actin* was used as the endogenous control.

### Cell Apoptosis Analysis by Flow Cytometery

After transfection for 36 h, cell apoptosis was analyzed by flow cytometry. The Annexin V-FITC Apoptosis Detection Kit (KeyGEN, Jiangsu, China) was used to stain the cells. Cells were harvested and wahsed twice with 1× PBS at a final concentration of 10^6^ cells per ml. Then, 500 μl cell suspension, 5 μl Annexin V-FITC conjugate and 10 μl propidium iodide solution were added into a test tube sequentially. The tubes were incubated at room temperature for 10 min at dark. Cells were subsequently analyzed by flow cytometry (BD, USA) to verify the effect of miR-152 and target genes on cell apoptosis.

### Cell Proliferation Assays

To determine the effects of miR-152 and target genes on MECs proliferation, miR-152, *ACAA2*, *HSD17B12* were overexpressed or silenced in MECs using the cell transfection methods individually. Briefly, cells (2 × 10^4^ per well) were seeded in 96-well plates. After transfection, proliferation was examined in the surviving fractions at 0, 12, 24, 36, 48 and 72 hours using the MTT assay (cellchipbj, Beijing, China). The absorbance was recorded at 450 nm using a micro-plate spectrophotometer (ACTGene, USA).

### Triglyceride Detection

Triglyceride was extracted from cells transfected with *miR-152 mimics*, *miR-152 inhibitor*, *miRNA-ShNC*, *PBI-CMV3-ACAA2/HSD17B12* and *sh234-ACAA2-181/sh234-HSD17B12-474* following the manufacturer’s instructions (Sigma, USA). Then the absorbance of the samples was detected with a SpectraMax M5 Microplate Reader (MD, USA).

## Electronic supplementary material

supplementary information
