# Supplementary material for: MiR-152 Regulates Apoptosis and Triglyceride Production in MECs via Targeting ACAA2 and HSD17B12 Genes
Source: Sci Rep. 2018 Jan 11;8:417. doi: 10.1038/s41598-017-18804-x (PMC5765104; doi:10.1038/s41598-017-18804-x)
Supplement: Supplementary file 1 — supplementary information [file 41598_2017_18804_MOESM1_ESM.pdf]

# MiR-152 Regulates Apoptosis and Triglyceride Production in MECs via Targeting ACAA2 and HSD17B12 Genes

Yuwei Yang[1,a] , Xibi Fang [1,a], Ruijun Yang[1], Haibin Yu[1], Ping Jiang[1], Boxing Sun[1,*],Zhihui Zhao[1,*]

[1] Agricultural College, Guangdong Ocean University, Zhanjiang 524088, China

[2] College of Animal Science, Jilin University, Xi An Road 5333, Changchun, Jilin 130062, P.R.China.

[a] They have the same contribution to the work and share the first author.

[*] Corresponding authors

Boxing Sun, sbx@jlu.edu.cn Tel./Fax: +86-431-87836189

Zhihui Zhao, a840581260@126.com Tel./Fax: +86-431-87836189

B-ACAA2



B-β-actin



B-HSD17B12



B-β-actin



**Fig. S1: Western blot analysis of the protein levels of *ACAA2* and *HSD17B12* genes in MECs transfected with *miR-152 mimics, miR-152 inhibitor* and *miR-shNC.***

The grouping of gels/blots cropped from different gels. The *ACAA2* and its' *β-actin* gels/blots were visualized under UV illumination with 3 min exposure time. The *HSD17B12* and its' *β-actin* gels/blots were visualized under UV illumination with 5 min exposure time.

ACAA2



β-actin



**Fig. S2: The proteins of *ACAA2* genes in MECs that transfected with *PBI-CMV3-ACAA2*，*PBI-CMV3*，*sh234-ACAA2-181*，*sh234* by western blot analysis.**

The grouping of gels/blots cropped from different gels. The *ACAA2* and its' *β-actin* gels/blots were visualized under UV illumination with 3 min exposure time.

HSD17B12 β-actin

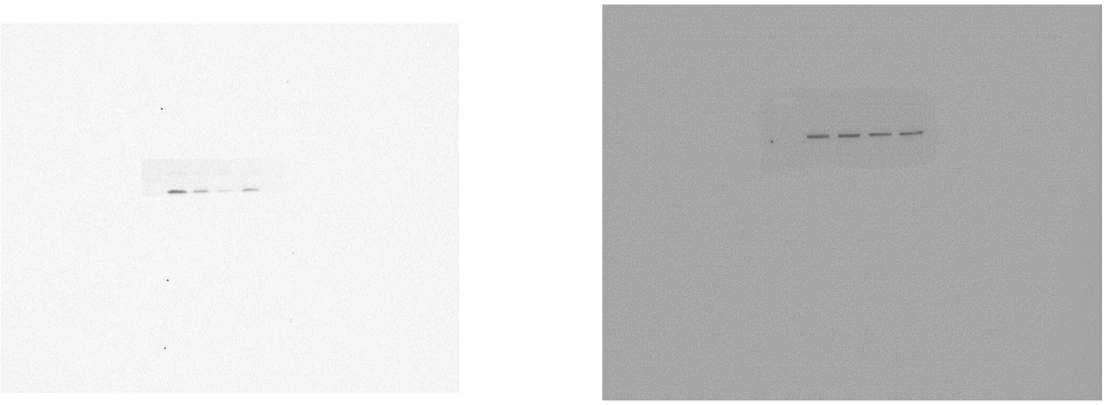

**Fig. S3 Western blot analysis of the protein levels of and *HSD17B12* genes in MECs transfected with *PBI-CMV3-HSD17B12*，*PBI-CMV3*，*sh234-HSD17B12-474*，*sh234***

The *HSD17B12* and its’ *β-actin* gels/blots were visualized under UV illumination with 5 min exposure time.
